# Growing Burden of Non-Communicable Diseases in the Emerging Health Markets: The Case of BRICS

**DOI:** 10.3389/fpubh.2015.00065

**Published:** 2015-04-23

**Authors:** Mihajlo B. Jakovljevic, Olivera Milovanovic

**Affiliations:** ^1^Department of Pharmacology and Toxicology, Faculty of Medical Sciences, University of Kragujevac, Kragujevac, Serbia; ^2^Department of Pharmacy, Faculty of Medical Sciences, University of Kragujevac, Kragujevac, Serbia

**Keywords:** health care, non-communicable disease, financing, sustainability, emerging market, BRICS, N-11

## Historical Perspective on Non-Communicable Diseases Worldwide

The blooming of incidence and prevalence of “prosperity diseases” among the broad layers of modern day populations is rather novel phenomenon in demographic history of the human race (1). Illnesses such as obesity (2), diabetes mellitus, hypertension, cerebrovascular and cardiovascular consequences of atherosclerosis, renal insufficiency, mental disorders, and even cancer are closely related to the increased longevity of most contemporary societies (3). In previous centuries, they were mostly reserved for elite social groups enjoying rather luxurious life style. Vast majority of citizens of the time were living in rural communities on the verge of poverty. Their structure of morbidity even in Europe until late 19th century was dominated by burden of infectious diseases and injury while neonatal and maternal mortality rates were huge. Industrial revolution led to the growth of living standards, invention of vaccines, and antibiotics, and ultimately development of organized publicly funded health systems. The prominent European health policy makers in the 19th century properly believed that effective public health measures will diminish huge burden of infectious diseases. Consecutively, they expected that overall costs of medical care provision should decrease substantially and ultimately reach plateau level. This second step turned out to be a great miscalculation and a surprise. Like no time in written past, people began living longer and healthier lives. But it happened at the cost. Simultaneously, from many industrialized nations, evidence were accumulating of accelerated occurrence of non-communicable diseases. Accomplishment of evidence-based medicine succeeded to control many of these initially incurable diseases, thereby transforming them into life time disorders as in the typical cases of diabetes and terminal renal insufficiency. Acute bacterial infections, dominating morbidity in the old days, were usually successfully treated within few weeks. Unlike these, chronic illnesses were bringing long-term burden for both the patients and the society. Malignant disorders with its complex treatment strategies present particularly demanding medical conditions. Cancer leaves permanent footprint in a life of a patient in terms of poor survival rates, decreased life quality, and working ability.

## Non-Communicable Diseases Expansion in Developing Countries

The ultimate demographic transition consisting of ascending portion of elderly, falling fertility rates, and bold growth of median age within contemporary nations became broadly recognized as population aging (4). Most of this transformation of morbidity and mortality structure happened in rich industrial countries of Western Europe, North America, and Japan many decades ago. The same pattern of population aging associated with huge incidence and prevalence rates of major non-communicable diseases repeated on wider scale much later in developing countries. The worldwide transformation of public health landscape to the large extent is attributable to the accelerated pace of globalization after the end of Cold War era (5). Particularly interesting, current developments belong to the economies responsible for most of global growth that are recognized as the emerging markets. The countries whose reshaped structure of morbidity is most likely to affect global health in the future are definitely the BRICS [Brazil (6), Russia, India, China, South Africa] (7). BRICS’s far extended long-term influence in health arena worldwide will be related to their mammoth sized populations. Their increased domestic demand for medical technologies and medicines is already shaping investment strategies of major pharmaceutical and medicinal device industries. Another significant issue is their bold foreign medical assistance programs particularly targeted for emerging markets of Sudanese Africa, Latin America, Central and South East Asia (8). These leading countries are closely followed by a set of smaller scale economies mostly marked as N-11 (Bangladesh, Egypt, Indonesia, Iran, South Korea, Mexico, Nigeria, Pakistan, the Philippines, Turkey, and Vietnam) (9). Very similar process is simultaneously taking place in dynamically developing Southern (10) and South-East Asian (11), Latin American, Eastern European (12), and Arab speaking MENA region (13). Eradication of poverty currently taking place in these regions is coupled with changed dietary habits (14) (higher salt and fat and lower carbohydrate intake), wide spread tobacco abuse, and sedentary life styles (15). The mentioned factors contributing to the growing burden of non-communicable diseases. It became obvious that contribution of emerging markets and Third World countries to the global economic burden of NCDs will grow further. It will, highly, likely, soon have greater share than the one of established mature market economies (16). As basic assumption of most forecasts remains the fact that such growth will be dominated by developments in China (17) and India (18). High toll of this unfortunate change for developing countries is coupled impact of communicable and non-communicable diseases (19). At the same time, many national health systems throughout Asia and beyond expose poor responsiveness to the NCDs related population needs. There seems to be serious barriers in access to medical care and its affordability to the ordinary citizens.

The increasing awareness on approaching of almost unbearable burden of NCDs (20) led to the high profile United Nations meeting on the subject in 2011 (21). Such UN gatherings are so uncommon on health related topics that it happened only once in past due to AIDS. NCDs recognized as the core global health challenges were cardiovascular disorders, cancer, diabetes, and chronic respiratory illness. These changes are beginning to profoundly change the landscape of even the poorest countries around the globe. So far, NCDs have already overarched burden of infectious diseases and injury in terms of disability adjusted life years, as well as work load and economic burden to the most national health sectors (22).

## Promising Cost-Effective Solutions for the Future

The blossoming of prosperity disease did not happen suddenly. It was a consequence of long chain of evolutionary events in civil society development. We will mention only some of them such as technological revolution, improved housing conditions, sanitation and sewage disposal, public health successes in eradication of major infectious diseases, policy efforts to tackle hunger and starvation among the world’s poor, and ultimately tobacco (23) and alcohol abuse (24). As its preconditions took so long to be created, it is unlikely that we shall be able to tackle NCD’s burden effectively in near future. Rich countries as well as developing ones concluded that orchestrated efforts will be needed in the international arena. World Health Organization has adopted a package of measures, whose implementation and progress are being monitored (25), broadly known as “Global coordination mechanisms on NCDs” (26). As most cost-effective and feasible measures were identified, control of tobacco consumption to the targeted 5% consumers worldwide until 2025 and reduction of salt intake by general populations of at least 15% in the order of significance. These interventions that were named “best buy” solutions offering best attainable compromise between the need for investment and outcomes that will be gained (27). Promotion of active life style and healthy diet, as well as other preventive and screening measures, comes at the second place. If such efforts are followed closely by national authorities, WHO expects that these measures should achieve 25% reduction of NCD attributable premature mortality until 2025 (28). Many of the proposed strategies were previously tested within a sound methodological framework applied on a second largest emerging market of the America, Mexico (29).

The most challenging issue for the emerging markets’ health systems appears to be universal health coverage (30). These systems were built up on diverse historical legacies and should find each one its own way to handle the upcoming pressure of prosperity diseases coupled with accelerated population aging. Profound transformation of current network of medical facilities in Third World countries, as well as human capacity building, will be forced to move priority from acute care toward complex, chronic illnesses (31).

## Growing Burden of NCDs Coincided with Increasing Health Expenditures

As witnessed by current WHO estimates given in Table 1, we may see that overall burden of non-communicable disease has consolidated in some countries such as Russia recording even slight decrease over the past decade. Nevertheless, leading emerging markets of China and India followed by a large distance in absolute terms by Brazil and South Africa exhibited clear pattern of increasing burden of NCDs expressed in terms of Years of Life Lost, Years Lost due to Disability, and Disability-Adjusted Life Year (DALY). According to WHO, NCDs attributable mortality increased substantially among the same four countries with notable promising exception of Russia. Russian partial success in containing but not decreasing toll of prosperity diseases over 2000–2012 observation period might be attributable to the strong public health legacy of Soviet era as well as reform policies implemented in recent past (32). The rates of hospital discharges increased substantially in the emerging markets across the globe following the increased presence of NCDs in the overall morbidity and mortality structure. This was mainly the case with clinical admissions that could be attributed to the malignant disorders (33) and circulatory diseases (34), followed by chronic obstructive pulmonary diseases (35) and diabetes (36). National level spending on medicines indicated to treat these conditions followed at the same pace, so entire regional pharmaceutical markets adjusted to these changes as was the case in South Eastern Europe (37). Extensive presence of chronic prosperity illnesses supported stronger demand for medical imaging (38), laboratory testing (39), outpatient visits, prescription and dispensing of novel pharmaceuticals (40), surgical, radiation oncology (41), and rehabilitation services. These phenomena were relying on strengthened civil expectations for advanced medical technologies supported by growing living standards and domestic consumption in BRICS markets. If we take into account serious challenge of home-based care for the disabled and growing portion of elderly citizens with special needs, bold growth of national health expenditures should have been predicted (42). China is absolutely leading in terms of purchase power parity of its health spending. Huge lag of all other major emerging economies behind People’s Republic of China is most obvious when compared to the India, rapidly developing nation of a similar population size.

**Table 1:** **Non-communicable diseases burden-related indicators; WHO estimates for BRICS in 2000 and 2012; total health expenditure and out-of-pocket health expenditure in terms of current international $ purchase power parity basis (source: Global Health Expenditure Database)**.

	Brazil		Russian federation		India		China		South Africa	
	2000	2012	2000	2012	2000	2012	2000	2012	2000	2012
Population (millions)	174.5	198.6	146.8	143.2	1,042.3	1,236.7	1,287.7	1,384.8	44.8	52.4
Years of Life Lost [YLL (′000)]*	22,532	24,915	44,566	40,597	150,751	175,435	165,905	186,591	5,534	7,398
Years Lost due to Disability [YLD (′000)]**	14,600	18,077	16,586	16,206	78,150	96,886	84,450	99,877	3,436	4,233
Disability-Adjusted Life Year [DALY (′000)]***	37,132	42,992	61,152	56,803	228,901	272,321	250,355	286,468	8,970	11,631
Estimated deaths (′000) NCDs caused, both sexes	777	978	1,819	1,801	4,579	5,869	6,839	8,577	176	264
Total expenditure on health (in million current $ PPP)	$87,681	$220,240	$54,200	$211,008	$68,816	$193,969	$138,131	$664,644	$24,728	$51,458
Out of pocket expenditure (in million current $PPP)	$33,277	$68,168	$16,242	$72,417	$46,771	$111,673	$81,469	$228,245	$3,227	$3,695

*WHO estimated Years of Life Lost (YLL) due to premature mortality NCDs caused, both sexes (′000).

**WHO estimated Years Lost due to Disability (YLD) for people living with NCDs or its consequences (′000).

***WHO estimated Disability-Adjusted Life Year (DALY) NCDs caused, both sexes (′000).

Catastrophic household expenditure presents particularly crucial issue throughout the countries of Sudanese Africa with very low incomes, whose medical care is dominantly supported by out-of-pocket spending (43). This happens due to absence of strong national health insurance funds whose revenues would come out of mandatory taxation supported by governmental and external financial sources. Huge, occasionally sevenfold growth of out-of-patient expenditure is clearly visible among the top BRICS markets. Such socioeconomic vulnerability seriously affects the poor members of the community. This might be the crucial issue for long-term affordability (44) of medical care to the ordinary citizens because almost all of the emerging markets own massive rural populations. Urbanization process, which began in Europe in 18th century, is still rapidly evolving throughout Asia, Africa, and Latin America (45). Extensive development of medical facilities network covering remote areas will remain one of the key difficulties for national governments. This is worsened by inevitable concentration of most professional staff in large cities with much more rewarding personal career opportunities. The primary goal for the future of these health systems wiil be provision of accessible medical care. It should have decent quality supported by universal health insurance coverage and full reimbursement of at least essential medicines. The speed of economic growth, political stability, and effectiveness of health reforms remain highly diverse among the top 20 emerging markets. Some global forecasting agencies as well as international financial organizations were pointing out that some smaller scale N-11 economies were top performers on some criteria. Nevertheless, the prevailing consensus is that BRICS (46) health care markets will inevitably outpace all others and remain well ahead of their competition shaping the global health challenges in the first half of 21st century.

## Conflict of Interest Statement

The authors declare that the research was conducted in the absence of any commercial or financial relationships that could be construed as a potential conflict of interest.
